# Complex Coding and Regulatory Polymorphisms in a Restriction Factor Determine the Susceptibility of *Drosophila* to Viral Infection

**DOI:** 10.1534/genetics.117.201970

**Published:** 2017-06-19

**Authors:** Chuan Cao, Rodrigo Cogni, Vincent Barbier, Francis M. Jiggins

**Affiliations:** * Department of Genetics, University of Cambridge, CB2 3EH, United Kingdom; † Institut de Biologie Moléculaire et Cellulaire, Centre National de la Recherche Scientifique, Strasbourg, 67084, France

**Keywords:** *Drosophila*, DCV, *pastrel*, viral infection, natural variation, Genetics of immunity

## Abstract

It is common to find that major-effect genes are an important cause of variation in susceptibility to infection. Here we have characterized natural variation in a gene called *pastrel* that explains over half of the genetic variance in susceptibility to the *Drosophila* C virus (DCV) in populations of *Drosophila melanogaster*. We found extensive allelic heterogeneity, with a sample of seven alleles of *pastrel* from around the world conferring four phenotypically distinct levels of resistance. By modifying candidate SNPs in transgenic flies, we show that the largest effect is caused by an amino acid polymorphism that arose when an ancestral threonine was mutated to alanine, greatly increasing resistance to DCV. Overexpression of the ancestral, susceptible allele provides strong protection against DCV; indicating that this mutation acted to improve an existing restriction factor. The *pastrel* locus also contains complex structural variation and *cis*-regulatory polymorphisms altering gene expression. We find that higher expression of *pastrel* is associated with increased survival after DCV infection. To understand why this variation is maintained in populations, we investigated genetic variation surrounding the amino acid variant that is causing flies to be resistant. We found no evidence of natural selection causing either recent changes in allele frequency or geographical variation in frequency, suggesting that this is an old polymorphism that has been maintained at a stable frequency. Overall, our data demonstrate how complex genetic variation at a single locus can control susceptibility to a virulent natural pathogen.

A central aim of infectious disease research is to understand why individuals within populations vary in their susceptibility to infection. This variation often has a substantial genetic component, and much effort has been devoted to identifying the genes involved [see reviews on humans (Burgner *et al.* 2006), plants (Alonso-Blanco and Mendez-Vigo 2014), and invertebrates (Obbard and Dudas 2014)]. It is common to find that natural populations contain major-effect polymorphisms that affect susceptibility to infection, especially when natural pathogens or parasites are studied. In humans, for example, major-effect genes affect susceptibility to *Plasmodium falciparum* malaria, *P. vivax* malaria, HIV, and Norwalk virus diarrhea (Hill 2012). Studying these genes can not only advance our understanding of the mechanisms of resistance and functioning of immune systems, but also provide insights into evolutionary processes. For example, theoretical models of host-parasite coevolution make strong assumptions about the genetic basis of resistance (Routtu and Ebert 2015). More generally, pathogens are one of the most important selective agents in nature, so understanding the genetic basis of how host populations respond to this selection pressure is of great interest.

While much research has focused on humans, crops, and domestic animals, studying the natural pathogens of model organisms such as *Arabidopsis*, *Drosophila*, and *Caenorhabditis elegans* provides a powerful way to understand the genetics of infectious disease resistance. There has been substantial research into genetic variation in susceptibility to viruses in *Drosophila*, with most research focusing on the sigma virus (Rhabdoviridae; DMelSV) (Longdon *et al.* 2012) and *Drosophila* C virus (DCV) (Dicistroviridae) (Johnson and Christian 1998; Hedges and Johnson 2008; Magwire *et al.* 2012; Kemp *et al.* 2013; Longdon *et al.* 2013; Zhu *et al.* 2013; Ferreira *et al.* 2014; Martins *et al.* 2014). DMelSV is a vertically transmitted virus that is relatively benign, causing an ∼20% drop in fitness (Yampolsky *et al.* 1999; Wilfert and Jiggins 2013). In contrast, DCV is horizontally transmitted and multiplies in most tissues of adult *Drosophila melanogaster*, causing marked pathogenic effects and sometimes death (Chtarbanova *et al.* 2014).

There is considerable genetic variation in susceptibility to both of these viruses within natural populations of *D. melanogaster* (Magwire *et al.* 2012). Much of this variation is caused by major-effect polymorphisms that confer a high level of resistance. In the case of DMelSV, three polymorphic resistance genes have been identified: *p62* [*ref*(*2*)*P*] (Contamine *et al.* 1989; Bangham *et al.* 2008), *CHKov1* (Magwire *et al.* 2011), and *Ge-1* (Cao *et al.* 2016). In a North American population, *p62* and *CHKov1* together explain 37% of the genetic variance in susceptibility to DMelSV (Magwire *et al.* 2011). Resistance to DCV is controlled by a very small number of genes, with a SNP in a gene called *pastrel* (*pst*) on chromosome 3 explaining 47% of the genetic variance in DCV susceptibility (Magwire *et al.* 2012). In another mapping population of flies, we recently reported that this gene accounted for 78% of the genetic variance (Cogni *et al.* 2016).

Despite its key role in virus resistance, *pst* remains poorly characterized. Its molecular function remains unknown, although it has been reported to participate in olfactory learning (Dubnau *et al.* 2003), protein secretion (Bard *et al.* 2006), and to be associated with lipid droplets (Beller *et al.* 2006). We identified the gene using an association study on 185 lines from North America with complete genome sequences (Mackay *et al.* 2012). In this study, six SNPs were found to be associated with resistance to DCV at *P* < 10^−12^, including two adjacent SNPs in the 3′ UTR (T2911C and A2912C), two nonsynonymous SNPs (G484A and A2469G), and two SNPs in introns (C398A and A1870G). All of these are in linkage disequilibrium (LD), and the nonsynonymous SNP A2469G in the last coding exon stands out as the most significant polymorphism (Magwire *et al.* 2012). However, the strong LD between SNPs prevents us from identifying the causal SNP(s).

In this study, we have characterized genetic variation in *pst* and its effects on susceptibility to viral infection. In a sample of seven copies of the gene from natural populations, we find four functionally distinct alleles that confer varying levels of resistance. By combining association studies and transgenic techniques, we identify an amino acid substitution that has led to a large increase in resistance. This appears to be a relatively old polymorphism that has been maintained at a relatively stable frequency in natural populations. The *pst* locus also contains complex structural variation and *cis*-regulatory variation affecting gene expression. Higher levels of *pst* expression are associated with increased resistance. Therefore, this is a complex gene in which multiple genetic variants affecting both gene expression and the amino acid sequence alter susceptibility to viral infection.

## Materials and Methods

### Generating transgenic flies carrying alleles of *pst* modified by recombineering

To test which SNPs in *pst* are causing flies to be resistant, we used recombineering to modify a bacterial artificial chromosome (BAC) clone of the region of the *Drosophila* genome containing the gene (Warming *et al.* 2005). This allowed us to make precise modifications of six candidate SNPs previously identified in *pst*, with five BACs carrying each SNP separately [SNP T2911C and SNP A2912C are adjacent and in complete LD in nature, so were considered as single locus TA2911(2)CC].

*Drosophila* P[acman] BACs were obtained from the BACPAC Resources Center (Venken *et al.* 2006, 2009). The *CHORI-322-21P14* clone, which covers a region of the fly genome that includes *pst* (genome positions: 3R: 2,114,276–21,164,956), was chosen for its smaller size (20.064 kb) and therefore higher transformation efficiency (Venken *et al.* 2009). This BAC does not contain any duplication or deletion of *pst*.

In the BAC clone containing *pst*, we modified the candidate SNPs controlling resistance using recombineering and *GalK* positive–negative selection following Warming’s protocol (Warming *et al.* 2005). *GalK*-targeting cassettes were PCR amplified from vector pgalK (Warming *et al.* 2005) using five different pairs of primers, each of which has ∼80 bp of sequence homologous to *pst* at each 5′ end. DNA fragments of size ranging from 300 bp to 1 kb that contained the SNP of interest were amplified using three *Drosophila* Genetic Reference Panel (DGRP) lines (Mackay *et al.* 2012) as template. Phusion High Fidelity polymerase (NEB) was used in the following conditions: 95° for 4 min; then 95° for 15 sec, 55° for 30 sec, and 68° for 1 min (1 min per 1 kb product), for 35 cycles; and incubate at 68° for 5 min. PCR products were gel purified using Invitrogen (Carlsbad, CA) PureLink Quick Gel Extraction Kit and fresh products were always used for transfection. Fly stocks used as template and primers used in PCR were listed in Supplemental Material, Table S1 in File S1.

We next inserted the five modified BAC clones containing the different *pst* alleles into identical sites in the genome of a fly line. This was possible as the BACs contain an *attB* site, which allows them to be inserted into *attP* docking sites of flies (Bischof *et al.* 2007). Plasmids of concentration between 0.1 and 0.3 μg/μl and an OD_260_/OD_280_ ratio between 1.8 and 1.9 were injected into the embryos of an *attP* line: *y*^−^*w*^−^*M*^(^*^eGFP^*^,^ *^vas-int^*^,^ *^dmRFP^*^)^*ZH-2A*;*P*{*CaryP*}*attp40*. Injected flies were crossed to a balanced *pst* hypomorphic mutant to generate line *w*;*transgenic*;*P^GSV1^GS3006/TM3*,*Sb^1^Ser^1^*. This balanced hypomorphic mutant has a *P* element inserted in the 5′ UTR of the *pst* gene and has a lower *pst* messenger RNA expression level than many laboratory fly stocks we tested. The fly crossing scheme is shown in Figure S1 in File S1.

### Overexpressing *pst* in flies

Transgenic flies that overexpress two different *pst* alleles were generated using vector pCaSpeR-hs fused with *pst* sequence. Expression of *pst* was under the control of the HSP70 promoter, and the protein is tagged by the FLAG epitope in the N terminus. The two *pst* alleles were amplified from complementary DNA from the fly lines DGRP-101 and DGRP-45 (Bloomington *Drosophila* Stock Center). These two DGRP lines encode identical *pst* amino acid sequences except for the Ala/Thr difference caused by SNP A2469G. Plasmids carrying different *pst* alleles were injected into a docker fly line containing an *attP* site on the second chromosome to form *y*^−^*w*^−^*M^eGFP^*^,^ *^vas-int^*^,^ *^dmRFP^ZH-2A*;*P*{*CaryP*}*attp40*. The experiment was subsequently repeated with a different fly line with a different *attP* site: *y*^−^*w*^−^*M^eGFP^*^,^ *^vas-int^*^,^ *^dmRFP^ZH-2A*;*M^attP^ZH-86Fb*. Male adults were crossed to the white-eye balancer *w^1118iso^/y^+^Y*;*Sco/SM6a*;*3^iso^* to select for successful transformants. Male and female transformants were crossed to generate homozygotes. The *attP* docker *y*^−^*w*^−^*M ^eGFP^*^,^ *^vas-int^*^,^ *^dmRFP^ZH-2A*;*P*{*CaryP*}*attp40* and the balancer used in crosses *w^1118iso^/y^+^Y*;*Sco/SM6a*;*3^iso^* were used as controls for measuring DCV mortality and viral titer. Two replicates (A and B) for each of the two *pst* alleles were established from independent transformation events. Western blot with FLAG antibody were carried out using adult flies that were kept in 25° to confirm the expression of FLAG-tagged *pst* alleles.

To assay the susceptibility of these lines to *pst*, vials were set up containing 10 females and 10 males of the transgenic lines and kept in 25°. The parental flies were removed and the progeny collected. A total of 15 vials containing 20 3- to 5-days-old mated females of each line were inoculated with DCV (or Ringer’s solution as a control) as describe below, and their mortality was monitored for 19 days. Meanwhile, 15 additional vials of each line with 15 mated females were inoculated with DCV and maintained at 25°. At day two postinfection, total RNA of these flies was extracted and used to measure viral RNA levels by quantitative real-time PCR (qRT-PCR) (see below).

### Measuring *pst* expression in DGRP lines

To study natural variation in gene expression, we measured *pst* expression in a panel of inbred fly lines from North America called the DGRP lines. We assayed 196 fly lines using one to seven biological replicates (a total of 654 RNA extractions). The flies were aged 6–9 days and a mean of 15 flies was used for each RNA extraction. These RNA extractions had been generated as part of a different experiment and were infected with Nora virus [*pst* is not associated with susceptibility to Nora virus (R. Cogni, personal communication) and expression of *pst* is not affected by Nora virus infection (Cordes *et al.* 2013)]. Primers and probes used are described below.

### Genotyping and naming of SNPs

DNA was extracted using either DNeasy Blood and Tissue Kit (QIAGEN, Valencia, CA) according to the manufacturer’s protocols or using a Chelex extraction which involved digesting fly tissues for 1 hr at 56° with 5% w/v Chelex 100 Ion Exchange Resin (Bio-Rad, Hercules, CA) in 200 μl of 33 mM dithiothreitol with 20 μg proteinase K (Jiggins and Tinsley 2005).

Diagnostic primers were designed to amplify the *pst* allele carrying specific SNPs. The SNP of interest was put at the 3′ end of one primer and at least one mismatch next to the SNP was introduced (Table S2 in File S1). To experimentally confirm the structural variants of *pst*, primers were designed to overlap the breakpoints of duplications and deletions (Table S2 in File S1). PCR products were run on 1% w/v agarose gels.

SNPs were named according to their position in the *pst* gene. Numbering begins at the nucleotide encoding the start of the 5′ UTR, and includes intronic positions. The numbering of duplications and deletions refers to the size of the region affected in nucleotides. In the text, we also report the genome coordinates of all variants.

### DCV

DCV stain C (Jousset *et al.* 1972) was kindly provided by Luis Teixeira (Teixeira *et al.* 2008) and was cultured in *D. melanogaster* DL2 cells using the protocol described in Longdon *et al.* (2013). The tissue culture infective dose 50 (TCID50) was calculated by the Reed–Muench end-point method (Reed 1938).

### Infection and resistance assay

Newly emerged flies were tipped into new food bottles. After 2 days, mated females were infected with DCV by inoculating them with a needle dipped in DCV suspension as described in Longdon *et al.* (2013) (TCID50 = 10^6^). Infected flies were kept on cornmeal food without live yeast on the surface. Numbers of infected flies that died were recorded every day and surviving flies were tipped onto new food every 3 days. Flies that died within 24 hr were excluded from the analysis as it was assumed that they died from the injection process. Infected flies were collected on day two postinfection for the measurement of viral titers.

### Quantitative real-time PCR

RNA was extracted using TRIzol (Invitrogen) in a chloroform-isopropanol extraction following the manufacturer’s instructions. RNA was used as template in qRT-PCR using QuantiTect Virus +ROX Vial Kit (QIAGEN). Dual-labeled probes and primers were ordered from Sigma-Aldrich. The PCR primers and probes amplifying both the reference gene and the gene of interest were multiplexed in a single PCR reaction. DCV titer was measured using probe DCV_TM_Probe ([6FAM]5′-CACAACCGCTTCCACATATCCTG-3′ [BHQ1]) and primers DCV_qPCR_599_F (5′-GACACTGCCTTTGATTAG-3′) and DCV_qPCR_733_R (5′-CCCTCTGGGAACTAAATG-3′). The amount of virus was standardized to a reference gene *RPL32* using probe Dmel_RpL32_TM_Probe ([HEX]ACAACAGAGTGCGTCGCCGCTTCAAGG[BHQ1]) and primers Dmel_RpL32_F (5′-TGCTAAGCTGTCGCACAAATGG-3′) and Dmel_RpL32_R (5′-TGCGCTTGTTCGATCCGTAAC-3′). Expression of *pst* was measured using dual-labeled probe Pst_PR ([Cy5]CAGCACACCATTGGCAACTC [BHQ3]) and primers Pst_FW (5′-CCGTCTTTTGCTTTCAATA-3′) and Pst_RV (5′-CCCAACTGACTGTGAATA-3′). The amount of *pst* expression was standardized to a reference gene *Ef1alpha100E* using the ΔΔCt (critical threshold) method (see below). Expression of *Ef1alpha100E* was measured using probe ([FAM] CATCGGAACCGTACCAGTAGGT [BHQ2]), primers Ef1alpha100E_FW (5′-ACGTCTACAAGATCGGAG-3′) and Ef1alpha100E_RV (5′-CAGACTTTACTTCGGTGAC-3′). Subsequent to the experiment, we realized there was a SNP segregating in the sequence to which the probe Pst_PR annealed, so the effect of this was corrected for by estimating the effect of this by linear regression and correcting the ΔCt values for its effect. This procedure did not qualitatively affect the conclusions. The estimation of gene expression or viral titer assumed that that the PCR reactions were 100% efficient. To check whether this assumption is realistic we used a dilution series to calculate the PCR efficiency. Three technical replicates of each PCR were performed and the mean of these was used in subsequent analyses. All the PCR efficiencies were between 97 and 103%.

### Statistical analysis of survival data and viral titers

R version 3.2.1 (R Development Core Team 2011) was used for statistical analyses. In the experiments using flies overexpressing *pst* and/or flies transformed with a modified BAC clone, we recorded the life span of individual flies. These data were analyzed with a Cox proportional hazard mixed model, fitted using the R package “coxme.” The genotype of the fly line was treated as a fixed effect. The random effects were the vial in which a fly was kept, which was nested in the replicate fly line (where the same fly genotype had been generated twice by independent transformation events). Flies alive at the end of the experiment were censored.

For each fly line in which we measured viral titers by qRT-PCR, we first calculated ΔCt as the difference between the cycle thresholds of the gene of interest and the endogenous controls (*actin 5C* or *Ef1alpha100E*). We used the mean values of technical replicates. To assess whether these differences were statistically significant, we fitted a general linear mixed model using the *lme* function in R. We used the mean ΔCt across all biological replicates as a response variable. The genotype of the fly line was treated as a fixed effect and the day that the flies were injected as a random effect.

### Identifying structural variants of *pst*

We identified structural variants by looking at the sequence data of the DGRP genomes (Mackay *et al.* 2012). Structural variants were detected when two halves of the same sequence read or read pair map to different positions or orientations within the reference genome. We analyzed 205 Freeze 2 BAM files of the DGRP lines (Mackay *et al.* 2012) using Pindel_0.2.0 (Ye *et al.* 2009) to identify the breakpoints of structural variants among the lines (deletions, tandem duplications, and large and small insertions). In 178 of the 205 lines (from which fly DNA was available), we confirmed the structural variants by carrying out PCR using primers either overlapping breakpoints or flanking them (Table S2 in File S1). We repeated this twice for the small number of lines that showed conflicting results with the Pindel analysis. We also Sanger sequenced the breakpoints in a subset of lines to confirm the predictions from the short-read analysis.

Duplications and deletions can also be detected by changes in sequence coverage. A script written in Python was used to calculate the coverage number for each base pair in the region 3L: 7,338,816–7,366,778 (BDGP 5).

### Identifying multiple alleles of *pst* with different effects on DCV susceptibility

We have previously measured survival after DCV infection in a panel of inbred fly lines called the *Drosophila* Synthetic Population Resource (DSPR) panel B (King *et al.* 2012a,b). These lines were constructed by allowing eight inbred founder lines with complete genome sequences to interbreed for 50 generations, and then constructing recombinant inbred lines (RILs) whose genomes were a fine-scale mosaic of these founders. We infected 619 RILs in panel B with DCV and monitored the mortality of 14,091 flies postinfection, which allowed us to identify *pst* as a major-effect gene defending flies against DCV infection (Cogni *et al.* 2016).

In this study, we reanalyzed this data set to test whether there were more than two alleles of *pst*. To identify different alleles of *pst*, we used a hidden Markov model (King *et al.* 2012a) to determine from which of the eight founder lines the *pst* allele had been inherited. We assigned RILs to one of the founders when position 3L: 7,350,000 (the location of *pst*) could be assigned to that parent with ≥95% confidence. We analyzed this data with a one-way ANOVA, with the mean survival time of each vial RIL as the response variable, and founder allele as a fixed effect. We then performed a Tukey’s honest significant difference test to assign the founders into allelic classes with differing levels of resistance.

### Identifying *cis*-regulatory polymorphisms in *pst*

To look for *cis*-regulatory polymorphisms that cause variation in *pst* expression, we used a set of microarray data of female head tissue in the DSPR (King *et al.* 2014). The mean normalized expression of three *pst* probes that did not contain any SNPs segregating in the panel (FBtr0273398P00800, FBtr0273398P01433, and FBtr0273398P01911) were used. The QTL analysis was performed using the R package DSPRqtl (http://FlyRILs.org/Tools/Tutorial) (King *et al.* 2012b) following Cogni *et al.* (2016).

### Association between *pst* expression and DCV resistance

To test whether the structural variants were associated with *pst* expression or susceptibility to DCV, we genotyped 178 DGRP lines for structural variants by PCR (primers listed in Table S2 in File S1). These variants were then combined with sequence data from the DGRP lines (Freeze 2). We have previously measured the survival of these fly lines after DCV infection (Magwire *et al.* 2012). The mean *pst* expression level was measured in 196 DGRP lines (see above). We then tested for associations between the SNPs in the region of 3L: 7,311,903–7,381,508 (BDGP5) and the mean of *pst* expression of each DGRP line using a linear model.

To estimate the genetic correlation between *pst* expression and survival after DCV infection in the DGRP lines, we used a bivariate general linear mixed model. The mean survival time of flies post-DCV infection was calculated for each vial assayed. *Pst* expression was measured on whole vials of flies. *pst* expression and survival were expressed as Gaussian response variables in the model:

$$y_{k,i,j}=t_{k}+b_{k,i}+\varepsilon_{k,i,j}\text{,}$$ (1)

where *y_i_*_,_*_j_*_,_*_k_* is the observed trait *k* (*pst* expression level or mean survival time) of flies from line *i* in vial *j*. *t_k_* is a fixed effect representing the mean expression level (ΔCt) or survival time. *b_ki_* are the random effects, which are assumed to be multivariate normal with a zero mean. For the random effects we estimated a two-by-two covariance matrix describing the genetic (between-line) variances of *pst* expression and survival, and the covariance between these traits. The genetic correlation was calculated from these parameters. *ε_k_*_;_*_i_*_;_*_j_* is the residual error, with separate residual variances estimated for the two traits. The parameters of the models were estimated using the R library MCMCglmm (Hadfield 2010), which uses Bayesian Markov chain Monte Carlo techniques. Each model was run for 1.3 million steps with a burn-in of 300,000 and a thinning interval of 100. Credible intervals on all parameters (variances, correlations, *etc*.) were calculated from highest posterior density intervals. The analysis was repeated including SNP A2469G as a fixed effect to control for any confounding effects of this variant being in LD with *cis*-regulatory polymorphisms (assuming this SNP is not itself a *cis*-regulatory polymorphism).

### Test for natural selection on *pst*

To investigate the frequency of resistance allele of A2469G in populations worldwide, we looked at publically available genome resequencing data sets of the Global Diversity Lines (Grenier *et al.* 2015), North American population (DGRP) (Mackay *et al.* 2012), and Zambian population [Drosophila Population Genomics Project (DPGP)] (Pool *et al.* 2012). We also collected 341 iso-female *D. melanogaster* from Accra, Ghana and genotyped a pool of flies from these lines for SNP A2469G by PCR as described above.

To test for a signature of natural selection on *pst*, we analyzed the sequence around *pst* from publically available genome sequences of *Drosophila*. These sequences were either from inbred lines or haploid genomes, so the data were phased as haplotypes. We analyzed data from two populations of *D. melanogaster* with large sample sizes: a North American population (DGRP, 205 lines) and a Zambian population (DPGP3, 197 lines). The variant calls from these lines in VCF file format of Freeze 2 DGRP was downloaded from the Baylor College of Medicine, Human Genome Sequencing Center Web site (Mackay *et al.* 2012). Because duplication and rearrangement of *pst* is very common in *D. melanogaster*, in the DGRP lines we Sanger sequenced *pst* from 35 lines of variant 3 and 28 lines of variant 4 so that we only analyzed data from the complete copy of the gene. These sequences were combined with 105 DGRP lines without rearrangement, resulting in a total of 165 DGRP lines with *pst* sequences. This was not possible for the data from Zambia as the original lines are not available. Here, consensus sequences of 197 *D. melanogaster* samples were downloaded from http://www.dpgp.org/. About 20-kb sequence around *pst* (3L: 7,340,375–7,363,363) were pulled out from all lines using the scripts “breaker.pl” and “dataslice.pl” written by the authors, returning FastA files. Then FastA file was converted into a VCF file by PGDSpider (Lischer and Excoffier 2012).

To examine how allele frequencies differ between populations, *F*_ST_ was calculated on a per-site basis for a North American population (DGRP) and a Zambian population (DPGP3) by VCFtools (Danecek *et al.* 2011). To detect LD around SNP A2469G, we estimated LD between all pairs of SNPs in a 20-kb region around it. The R packages “genetics” and “LDheatmap” (Shin *et al.* 2006) were used to calculate and plot LD in a heat map. We then applied the long-range haplotype test (Sabeti *et al.* 2005; Zeng *et al.* 2007) to examine the extended haplotype homozygosity (EHH) around SNP A2469G in comparison with other haplotypes of similar frequency in the 200-kb region (3L: 7,250,375–7,253,363). The R package “rehh” was used in the analysis (Gautier and Vitalis 2012).

We finally applied a McDonald and Kreitman test (MKT) to detect positive selection on the amino acid level (McDonald and Kreitman 1991). Using the *D. yakuba* sequence as an outgroup, substitutions were polarized along the lineage leading from the common ancestor of *D. melanogaster* and *D. simulans* to *D. melanogaster*. A standard MKT was carried out using MKT software (Egea *et al.* 2008). We excluded polymorphic sites with a frequency of <10% to reduce the number of deleterious amino acid polymorphisms in the data set. Polarized two-by-two contingency tables were used to calculate α, which is an estimate of the proportion of amino acid substitutions fixed by selection (Smith and Eyre-Walker 2002). Statistical significance of the two-by-two contingency tables was determined using a χ^2^ test.

Nucleotide diversity was calculated using DnaSP version 5 (Librado and Rozas 2009) for the 20-kb region described above in 165 DGRP lines and 197 DPGP lines.

### Data availability

For fly stocks and primers see Table S1 and Table S2 in File S1. The raw data and scripts used in this study are available in the University of Cambridge data repository at http://dx.doi.org/10.17863/CAM.866.

## Results

### The *pst* locus has multiple alleles affecting DCV resistance

**Figure 1:**
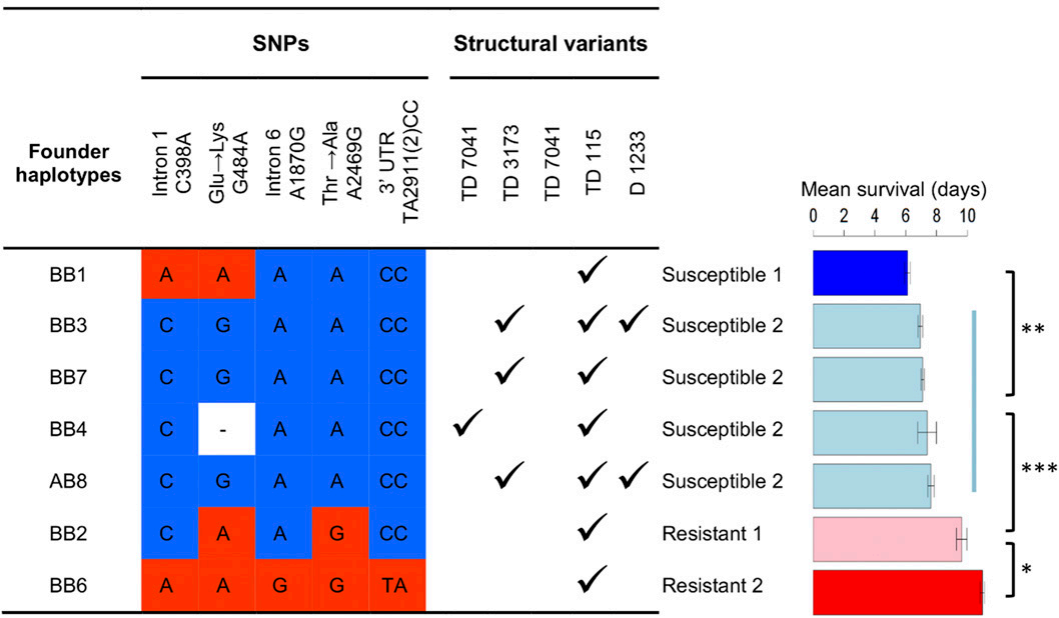
Genetic variation and susceptibility to DCV of the *pastrel* haplotypes segregating in the DSPR panel. The genotypes of SNPs that were strongly associated with resistance in a previous association study are shown (Magwire *et al.* 2012). Susceptible SNPs are in blue and resistant SNPs are in red. We estimated the mean survival time of the founders using an ANOVA, and identified groups of founder haplotypes with significantly different levels of resistance using Tukey’s honest significant differences test. There are four phenotypically distinct classes of alleles that have significantly different effects on resistance (* *P* < 0.05, ** *P* < 0.01, *** *P* < 0.001). In total, the survival of 13,919 flies was analyzed. Error bars are SEs. The locations of the SNPs in chromosome 3L of the *Drosophila* genome are C398A: 7352966; G484A: 7352880; A1870G: 7351494; A2469G: 7350895; TA2911(2)CC: 7350452-3. The structural variants are named according to whether they are tandem duplications (TD) or deletions (D).

In a previous association study we found six SNPs in *pst* that were strongly associated with DCV resistance (Magwire *et al.* 2012). All of these are in LD with each other, so it was not possible to identify the causative variant from these data. Intriguingly, however, no single SNP could explain all the effects of *pst* on DCV susceptibility, suggesting that multiple alleles of this gene with different susceptibilities might be segregating in populations. To investigate this further, we reanalyzed a second data set where we had infected 13,919 flies from the DSPR [panel B, 619 RILs founded by eight lines representing a worldwide sample (King *et al.* 2012b)] with DCV and shown that resistance was largely controlled by *pst* (Cogni *et al.* 2016). These data allow us to estimate the effect that each of the seven different founder haplotypes of *pst* segregating among these lines has on DCV susceptibility (one of the eight founders, BB5, was removed from analysis because it is represented by <10 lines and was not able to be assigned to any group). The seven founder haplotypes fall into four groups with significantly different resistance levels (Figure 1). Flies in the resistant 1 (resist1) group survived an average of 9.6 days postinfection while flies in the resistant 2 (resist2) group survived an average of 11 days postinfection. Flies in the susceptible 1 (susc1) group survived an average of 6.1 days postinfection while flies in the susceptible 2 (susc2) group survived an average of 7.1 days postinfection (Figure 1). Therefore, in a sample of seven copies of this gene, there are four functionally distinct alleles of *pst* affecting DCV resistance.

### The amino acid substitution A2469G can explain resistance in two different genetic mapping experiments

We examined the six *pst* SNPs previously found to be associated with resistance in our genome-wide association study in DGRP lines (*P* < 10^−12^) and asked which of them explain the four levels of resistance we observed in the DSPR founders. Only A2469G, which is a nonsynonymous change (Thr/Ala, 3L: 7,350,895, BDGP5), can explain the large difference between the two resistant and the two susceptible classes of alleles (Figure 1). This change is also the most significant SNP in the association study using the DGRP lines (Magwire *et al.* 2012) and in a separate study that had selected populations for DCV resistance and then sequenced their genomes (Martins *et al.* 2014). This threonine to alanine change is a radical substitution between a polar and a nonpolar amino acid, and alanine is associated with increased resistance in both the association study and this QTL analysis. Two closely related species, *D. simulans* and *D. yakuba*, both have a threonine at this position; indicating that the susceptible allele was the ancestral state. While this analysis strongly implicates A2469G in resistance, it does not preclude a role for the other five variants associated with resistance. For example, SNP C398A differs between the susc1 and susc2 alleles, while SNPs TA2911(2)CC, A1870G, and C398A all differ between resist1 and resist2.

### Modifying SNP A2469G in transgenic flies confirms that it alters resistance to DCV

To experimentally confirm the SNP(s) causing flies to be resistant to DCV, we generated five transgenic lines where we modified each of the six SNPs associated with resistance [SNP T2911C and A2912C, which are in complete LD, were modified together: TA2911(2)CC]. To do this we edited a BAC clone (*CHORI-322-21P14*, 20.064 kb) of the region in *Escherichia coli*. The BAC originally contains the allele associated with increased resistance for all five of the *pst* variants, and we individually changed these to the susceptible variant. We inserted the five BACs into the same genomic position in a fly line to generate five transgenic lines. We crossed these transgenic flies to a balanced *pst* hypomorphic mutant *y^1^w^67c23^*;*If/Cyo*;*P^GSV1^GS3006/TM3*,*Sb^1^Ser^1^*, which has a transposable element inserted in the 5′ UTR of the *pst* gene. The transgenic alleles did not complement the lethal effect of this mutation upstream of *pst*, so we infected flies that were homozygous for the transgenic *pst* allele on chromosome 2 and had one hypomorphic mutant allele over a balancer chromosome on chromosome 3 (*pst* hypomorphic mutant allele and the balancer carries the susceptible form “A” for SNP A2469G).

**Figure 2:**
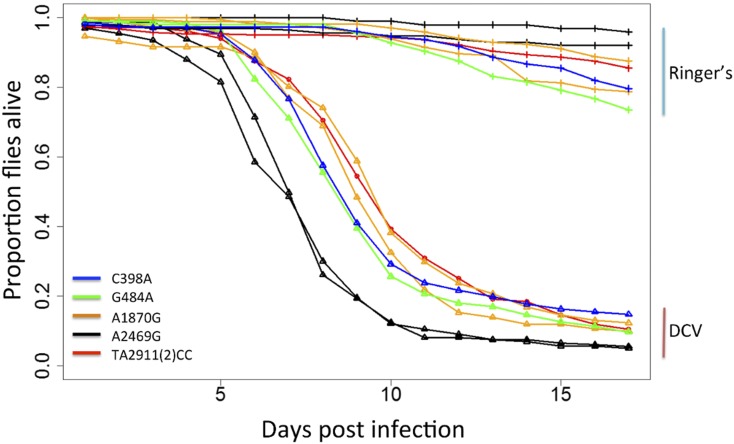
Susceptibility to DCV in transgenic flies carrying different alleles of *pst* SNPs. Lines with ▴ are flies infected with DCV while lines with + are flies injected with Ringer’s solution as a control. SNP A2469G and SNP A1870G have two biological replicates, which were generated through independent transformation events. By fitting a Cox proportional hazard mixed model, we found that A2469G is significantly different from all the other SNPs (*P* < 0.007). There were no significant differences among the other four SNPs (*P* > 0.36). In total, 157 vials containing 3010 females were infected with DCV and their mortality were recorded daily for 17 days.

The two independent fly lines carrying an A for SNP A2469G, which were generated through independent transformation events, died significantly faster after DCV infection compared with all the other transgenic lines that had a “G” at this position (Figure 2). There were no significant differences among the other four genotypes. Among the flies that were mock infected with Ringer’s solution, there were no significant differences among lines (although flies carrying a susceptible A at A2469G survived longest, which reinforces the result that the high mortality of these flies when DCV infected is being caused by *pst*). In summary, both genetic mapping approaches and experimentally modifying the gene demonstrate that the SNP A2469G is causing flies to be resistant to DCV.

### Overexpressing both the resistant and susceptible alleles of *pst* protects flies against DCV infection

**Figure 3:**
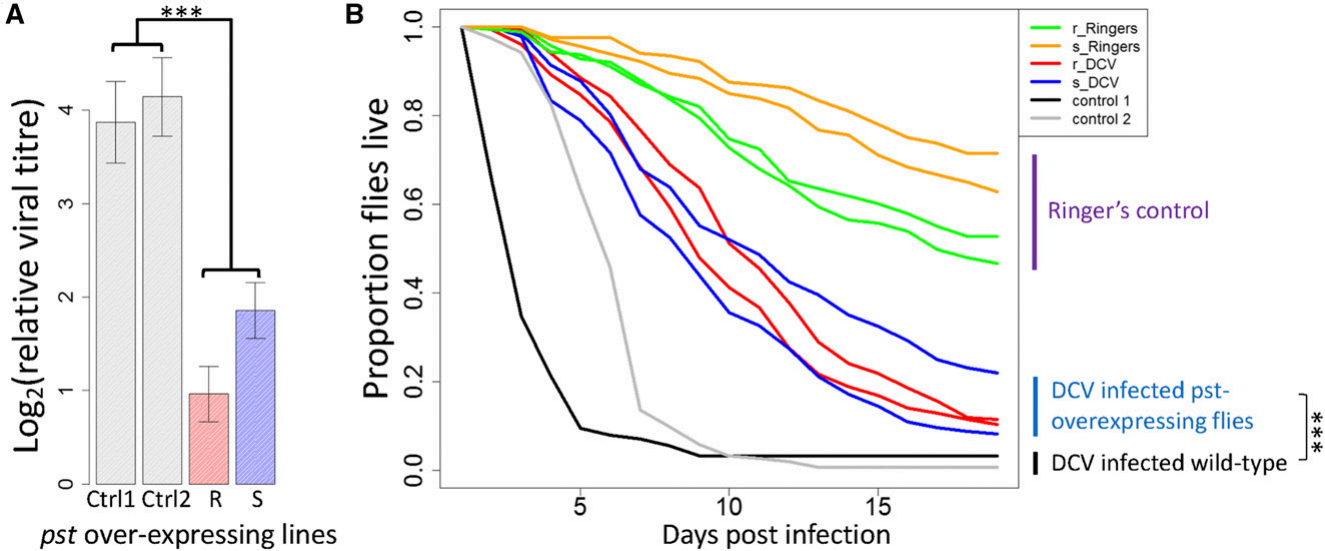
The effect of overexpressing *pst* carrying the susceptible (s) and resistant (r) alleles of SNP A2469G on survival and viral titer. (A) DCV titer relative to *Act5C* in flies 2 days postinfection. Bars are the means of 28 vials each containing 15 flies. Error bars are SEs. (B) The proportion of flies alive after infection with DCV or mock infection with Ringer’s solution. The survival curves are the mean of ∼15 vials of flies, with a mean of 18 flies in each vial. Flies were kept at 25°. Control 1 (Ctrl1) were docker flies into which the BAC constructs were inserted (*y*^−^*w*^−^*Me^GFP^*^,^ *^vas-int^*^,^ *^dmRFP^ZH-2A*;*P*{*CaryP*}*attp40*), and control 2 (Ctrl2) were flies used in the crosses to select successful transformants (*w^1118iso^/y^+^Y*;*Sco/SM6a*;*3^iso^*). The experiments used two independent transformants of each construct (A and B). *** *P* ≪ 0.001.

Resistance could evolve by altering host factors that are beneficial to the virus or by increasing the efficacy of existing antiviral defenses. To distinguish between these hypotheses, we generated fly lines that overexpress either the resistant or the susceptible allele of *pst* (these constructs encode a protein that only differs at the site affected by SNP A2469G). The two FLAG-tagged constructs were inserted at the same *attp40* of the fly genome using *phiC31* integrase, and we checked that the full-length protein (∼77 kDa) was being expressed using a Western blot targeting the FLAG tag. Two replicates of these lines were generated and these flies were then infected with DCV. We found that overexpressing both the susceptible and the resistant alleles of *pst* led to significant reductions in viral titers at 2 days postinfection (Figure 3A; general linear model: *pst*_A_: |*z*| = 3.3, *P* = 0.003, *pst*_G_: |*z*| = 4.83, *P* < 0.001). There is no significant difference in viral titer between flies overexpressing the resistant *pst* allele G and flies overexpressing the susceptible *pst* allele A, although the trend is in the expected direction (Figure 3A; |*z*| = 1.4, *P* = 0.35). Next, we examined survival. Overexpressing *pst*, no matter which allele, substantially increased survival after DCV infection (Cox proportional hazard mixed models; *pst_A_*: |*z*| = 12.32, *P* < 1*e*^−5^, *pst_G_*: |*z*| = 11.83, *P* < 1*e*^−5^) (Figure 3B). Again, we were not able to detect any difference in mortality between flies overexpressing the two different alleles of *pst* (|*z*| = 0.53, *P* = 0.86). This result should be interpreted with caution, as any differences in resistance between the two alleles may be obscured by intrinsically lower survival of the flies overexpressing the resistant allele (Figure 3B, Ringers control). This difference in the survival of mock-infected flies overexpressing the different alleles could not be replicated when new transgenic flies were generated in a different genetic background and assayed without pricking, suggesting that it is not a toxic effect of the resistant allele (Figure S2 in File S1). In summary, overexpressing either *pst* allele substantially increased resistance, with the resistant allele causing a slightly greater reduction in viral titer.

### The *pst* locus contains complex structural polymorphisms

**Figure 4:**
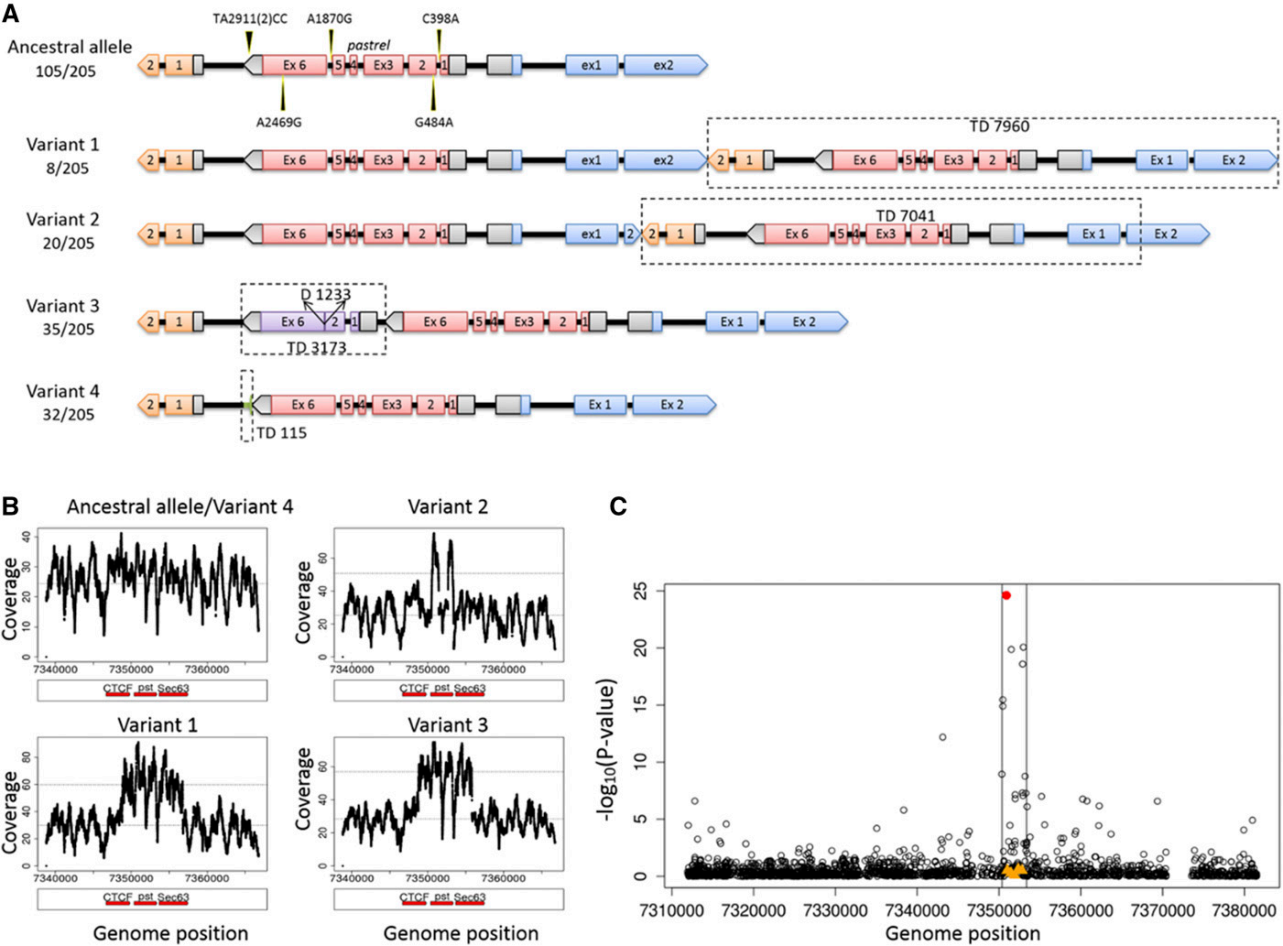
Five structural variants of *pastrel*. (A) Cartoon of *pst* variants, with alleles’ size scaled to gene length. Pink boxes represent complete copy of *pst* gene; orange boxes represent coding sequence of gene *CTCF* located at 3′ end of *pst*; blue boxes represent coding sequence of gene *Sec63*, located at 5′ end of *pst*; gray boxes are UTRs; purple boxes are truncated copy of *pst* gene. Allele frequencies in DGRP are shown below the variant name. Variant 2 differs from variant 1 in that variant 2 has a shorter duplication of CTCF exon (ex) 2. (B) Mean sequencing coverage plots of the region 3L: 7,338,816 (1-kb upstream of the start of TD7960)–7,366,778 (1-kb downstream of the end of TD7960) for ancestral allele of *pst* and four structural variants. Red bars stand for *pst* and two neighbor genes *CTCF* and *Sec63*. Variant 4 has a very short duplication of 115 bp so shows very similar coverage plot as the ancestral allele. Sequence data are from the original DGRP genome sequencing project (Mackay *et al.* 2012). (C) Association between survival after DCV infection and *pst* SNPs and structural variants. −Log_10_(*P*-value) of the association between SNPs in the region of 3L: 7,311,903–7,381,508 (BDGP 5), and survival is plotted against genome positions of the SNPs. SNPs are showed as ○; SNP A2469G is in red. Structural variants of *pst* are showed in orange ▴’s.

The analyses above only considered SNPs, but other types of genetic variation could cause flies to be resistant. We therefore investigated the existence of structural variation in a panel of 205 inbred fly lines from North America whose genomes had been sequenced (DGRP) (Mackay *et al.* 2012). The existence of structural variation had been suggested by the PCR amplification of a truncated copy of *pst* in certain flies and cell lines. We identified the breakpoints of structural variants from published paired-end, short-read sequencing data (using Pindel_0.2.0) (Ye *et al.* 2009). Excluding small indels, this approach revealed five variants that were shared by more than three lines and supported by at least four raw sequencing reads (Figure 4A; the region investigated, 3L: 7,346,678–7,357,466, DPGP 5, includes *pst* and the two flanking genes *CTCF* and *Sec63*). In 178 of the 205 lines we confirmed the structural variants by carrying out PCR with diagnostic primers and Sanger sequencing. As a final confirmation, we checked that the duplicated regions had increased sequence depth (Figure 4B).

The five major structural variants and their frequencies in the DGRP lines are summarized in Figure 4A. Just over half of the lines had the ancestral state which is found in the reference genome with one complete copy of *pst* (Figure 4A; ancestral allele). A total of 8 out of 205 lines have a 7960-bp duplication (3L: 7,348,816–7,356,777, variant 1) containing a complete copy of *pst* and some sequences from two adjacent genes (*CTCF* and *Sec63*). A total of 20 lines have a 7041-bp duplication (includes *pst* and partial sequences from neighbor genes, 3L: 7,348,778–7,355,820, variant 2). Additionally, 35 lines have a duplicated copy of *pst* (3L: 7,350,246–7,353,420) with a 1233-bp deletion in the middle (variant 3). Finally, 32 lines have a duplication of 115 bp (3L: 7,350,263–7,350,379) at the 3′ end of *pst* (variant 4). There are another five lines containing structural variants each represented by less than three lines that are not shown in Figure 4A.

We tested whether these structural variants affect survival of the DGRP lines after DCV infection and found that none of the structural variants is associated with survival post-DCV infection (*F*_1,165_ < 1.97, *P* > 0.32; Figure 4C). This nonsignificant result may due to a lack of power. For example, one of the structural variants that has a complete copy of *pst* was only represented by as few as eight lines. Another possible explanation is that transcripts produced by these duplicates are nonfunctional.

### There is *cis*-acting genetic variation that alters the expression of *pst*

**Figure 5:**
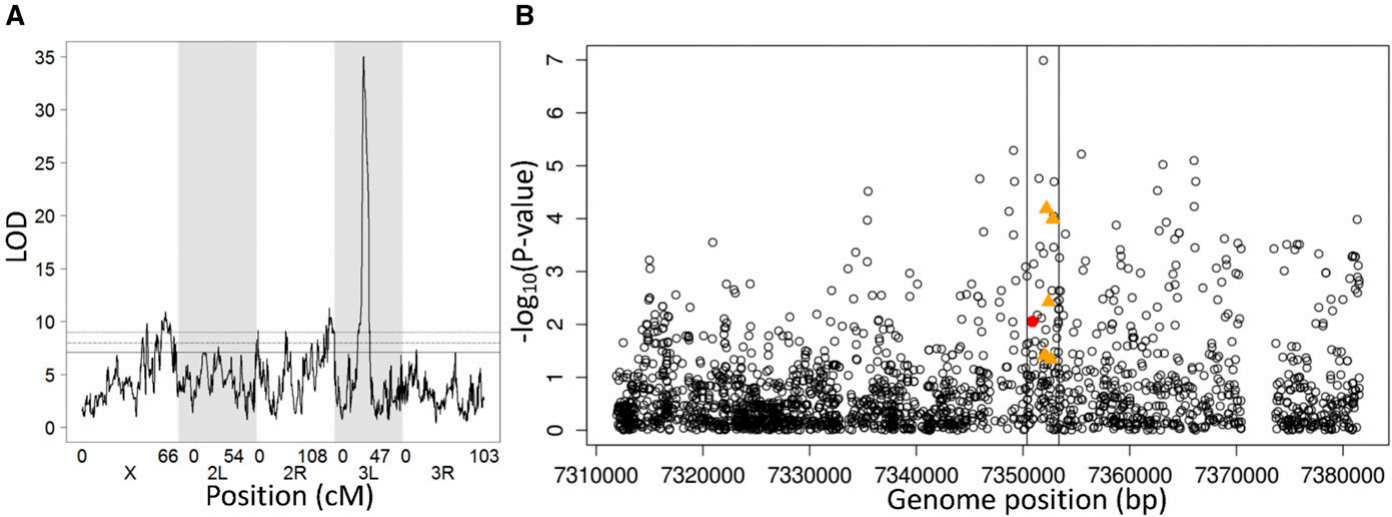
*Cis*-regulatory variation in *pst* expression. (A) Map of QTL associated with *pst* expression in female head of DSPR crosses. A single peak at position 3L: 7,350,000 was found (LOD = 35). The horizontal line is the genome-wide significance threshold obtained by permutation (*P* < 0.05, LOD = 7.12). Expression data are from published microarray analysis (King *et al.* 2014). (B) Association between *pst* expression and its SNPs and structural variants in DGRP lines. Gene expression was measured by qRT-PCR on 654 biological replicates of 196 fly lines. −Log_10_(*P*-value) for the association between SNPs and expression in the region of 3L: 7,311,903–7,381,508 (BDGP 5) is plotted against genome positions of the SNPs. SNPs are showed as ○; SNP A2469G in red. Structural variants of *pst* are showed in orange ▴’s.

Given that altering the expression of *pst* experimentally alters resistance to DCV, it is possible that natural variation in gene expression affects susceptibility to the virus. We investigated this using published microarray data from F_1_ individuals from crosses between two panels of recombinant fly lines derived from 15 founder lines from around the world (crosses between DSPR panel A females and panel B males) (King *et al.* 2014). To map regions of the genome affecting *pst* expression, we used the mean normalized expression of three *pst* probes (FBtr0273398P00800, FBtr0273398P01433, and FBtr0273398P01911) that did not contain any SNPs. We found that there was a major QTL controlling *pst* expression at 3L: 7,350,000 (LOD = 35.04), which is very close to the location of *pst* (Figure 5A). Therefore, there is genetic variation in *pst* expression and this is controlled by *cis*-acting genetic variants close to *pst* rather than variation elsewhere in the genome acting in *trans*.

To investigate which genetic variants might be affecting *pst* expression, we measured expression across 198 DGRP lines using qRT-PCR. Using these data, we looked for associations between the five structural variants and SNPs in the region surrounding *pst* (Figure 5B). We found *pst* expression was most significantly associated with a SNP in an intron of *pst* at position A1455T (3L: 7,351,909, *F*_2,155_ = 17.89, *P* = 1.02*e*^−7^). However, several of the structural variants were also associated with *pst* expression (Figure 5B). Tandem duplications TD3173 and TD115 were in LD with SNP A1455T (Fisher’s exact test: *P* = 0.002 and *P* = 0.001), but they remain significantly associated with *pst* expression after accounting for SNP A1455T by including it as a covariate in the model (TD3173: *F*_1,156_ = 14.07, *P* = 0.0002; TD115: *F*_1,156_ = 7.7, *P* = 0.006; Figure 5B). TD3173 is in strong LD with D1233 (Fisher’s Exact Test, *P* < 2.2*e*^−16^). Therefore, multiple *cis*-regulatory variants affect *pst* expression, and these may include structural variants.

### The expression of *pst* is correlated with DCV resistance

**Figure 6:**
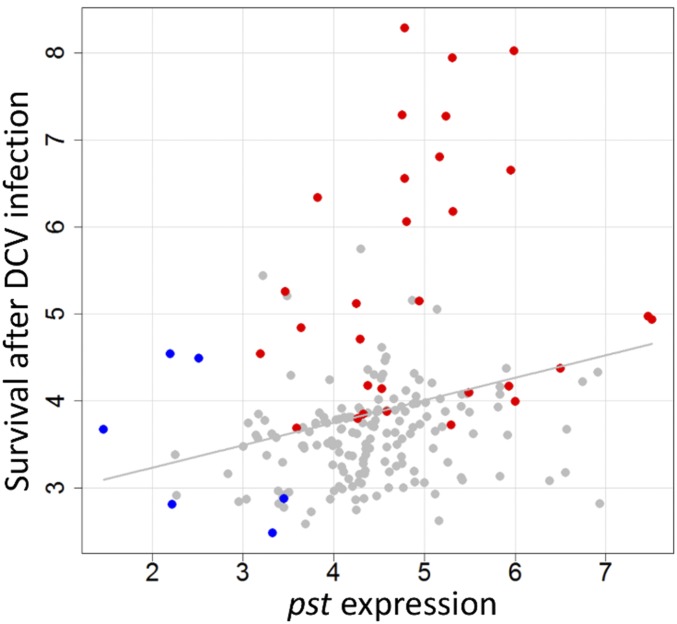
Correlation between *pst* expression and survival after DCV infection in DGRPs. Gray line is fitted by linear regression line and is shown for illustrative purposes only. Each point is the estimated phenotype of a single DGRP line (marginal posterior modes of the random effects in model Equation 1). Red ●’s represent lines that contain resistant allele G for SNP A2469G and blue ●’s represent lines contain “T” for SNP A1455T. Gene expression was measured by qRT-PCR on 654 biological replicates of 196 fly lines. Survival after DCV infection was estimated from 730 vials of flies, with the data from Magwire *et al.* (2012).

Across 198 DGRP lines we found that natural variation in *pst* expression was correlated with survival after DCV infection (Figure 6; genetic correlation: *r*_g_ = 0.32, 95% C.I. = 0.17–0.45). This is consistent with previous results that DCV resistance changes when *pst* is knocked down by RNA interference (RNAi) (Magwire *et al.* 2012) or overexpressed using transgenic techniques (Figure 3). SNP A2469G is a nonsynonymous SNP that was found to affect survival after DCV infection, which means it is unlikely to have an effect on gene expression. However, if it is in LD with a *cis*-regulatory variant, this could create spurious associations between gene expression and resistance. To control for this, we estimated the correlation after accounting for the effect of SNP A2469G by including it as a covariate in the model, and found the correlation between *pst* expression and survival after DCV infection remains significant (genetic correlation: *r*_g_ = 0.25, 95% C.I. = 0.11–0.41). These results indicate that *cis*-regulatory variation that alters *pst* expression and affects resistance to DCV.

### There is no evidence of spatially varying selection acting on the resistant allele of *pst* A2469G

Having identified the genetic variant that is responsible for most of the genetic variation in DCV resistance in *D. melanogaster*, we are well placed to characterize how natural selection has acted on this variant. It is common to find that the prevalence of viruses in *Drosophila* varies geographically (Carpenter *et al.* 2012; Webster *et al.* 2015), and this is expected to result in spatially varying selection pressure for resistance. However, there is little variation in the frequency of the resistant allele between populations. The resistant allele of A2469G is at a low frequency in populations worldwide: 7.7% in Zambia [197 DPGP3 lines (Pool *et al.* 2012)], 16% in North America [205 DGRP lines (Mackay *et al.* 2012)], 10% in Beijing [15 GDL lines (Grenier *et al.* 2015)], 5% in The Netherlands (19 GDL lines), 33% in Tasmania (18 GDL lines), and 10% in Ghana (341 lines collected and genotyped in this study). Among the populations with genome sequence data, only Zambian and North American populations have large sample sizes (197 lines and 205 lines, respectively), so the following analyses were carried out on these two data sets.

**Figure 7:**
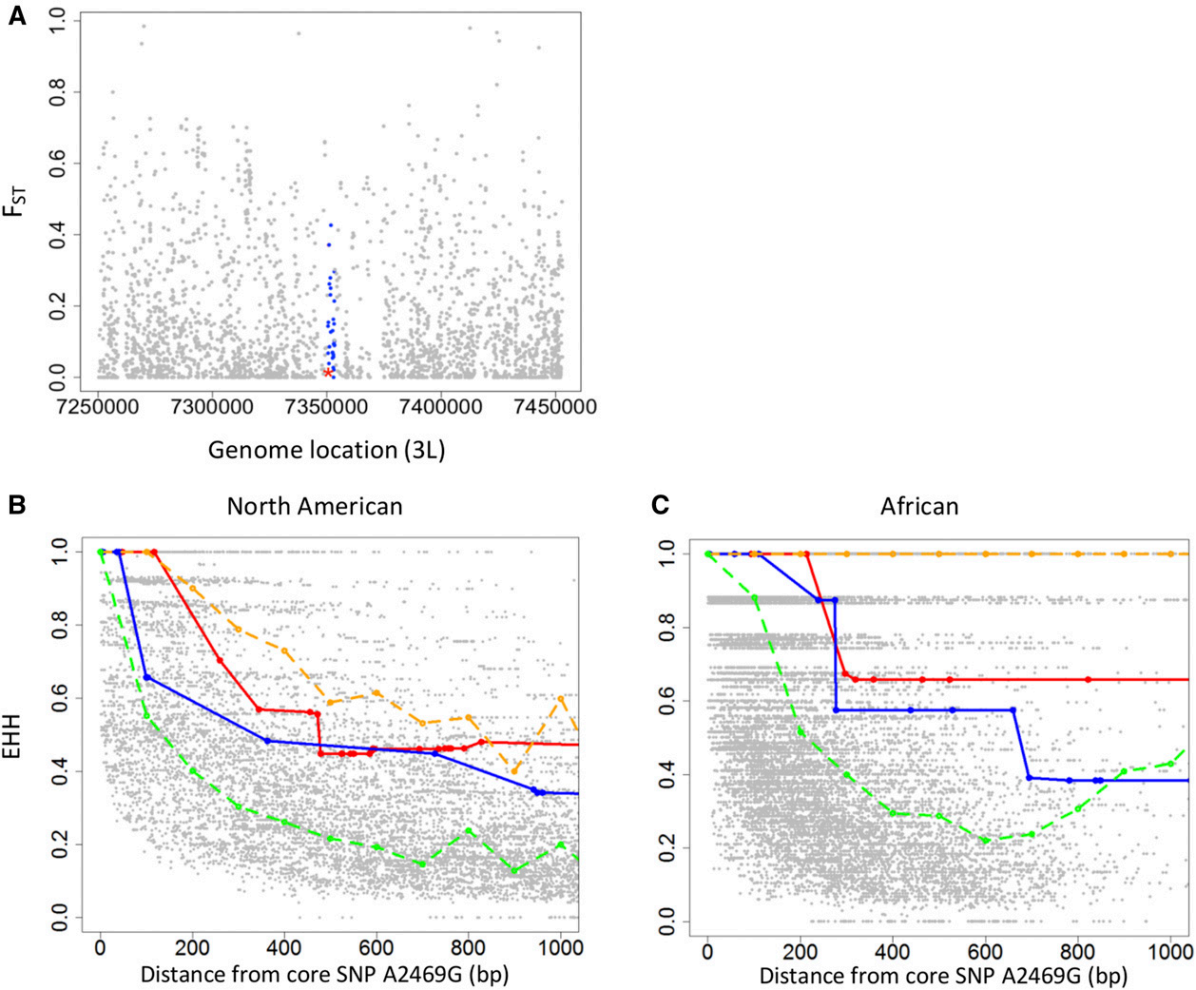
Population genetic analysis of natural selection acting on the amino acid polymorphism A2469G in *pst* that confers resistance to DCV. (A) *F*_ST_ of all SNPs within 200-kb region around *pst*. Blue ●’s are SNPs in *pst*, and the red * is A2469G. *F*_ST_ was calculated between Zambia and North America using published genome sequences (see text). (B and C) The breakdown of EHH over distance between the derived (resistant) allele of the core SNP A2469G and SNPs within the distance of 1000 bases from the mutation are shown. Red line and blue line are EHH breakdown upstream and downstream of SNP A2469G, respectively. The gray points are a null distribution generated by calculating the EHH using other SNPs that are a similar frequency in the region as the core. The orange dash line indicates top 5% EHH value of this null distribution while green dash line indicates median EHH.

To compare the geographical variation in allele frequency at A2469G to other SNPs in the region, we calculated *F*_ST_ (a measure of differences in allele frequency) between North America and Zambia. It is clear that A2469G (red * in Figure 7A) is not a significant outlier relative to the other 2641 SNPs analyzed in the 100-kbp region on either side (SNPs that have a minor allele frequency <5% were filtered out), indicating there is no evidence of population-specific selective pressure on SNP A2469G.

### The resistant allele of *pst* is old and shows no evidence of recent changes in frequency driven by natural selection

When natural selection causes an unusual, rapid rise in allele frequency, there is little time for recombination to break down the haplotype carrying the selected mutation. This results in unusual long-range haplotypes and elevated LD around the variant given its population frequency. As we know the site that is likely to be a target of selection, this is a powerful way to detect the effects of selection on DCV resistance. We first measured the LD between SNP A2469G and SNPs in a 10-kb region upstream and downstream of it. In both Africa and North America we found very little LD between SNP A2469G and surrounding SNPs (Figure S3, A and B, in File S1).

When the variant under selection is known, the most powerful test for such effects is the EHH test (Sabeti *et al.* 2005; Zeng *et al.* 2007). We calculated the EHH using the resistant (derived) allele of SNP A2469G as a core, and compared this to a null distribution generated from other SNPs of similar frequency that were nearby in the genome (Figure 7, B and C). In both populations, although the EHH around the resistant allele of A2469G is above the median, it is below the top 5%. Therefore, there is no evidence of positive selection on the resistant allele of A2469G generating extended LD around this variant. We also calculated the EHH for the susceptible allele of SNP A2469G as a core, and found no extended LD around this variant (Figure S4 in File S1).

Positive and balancing selection can also affect the nucleotide diversity (π). In a 20-kb region around *pst* in both North American and African populations, we did not observe elevated nucleotide diversity compared to the π value of the whole genome (Figure S5, A and B, in File S1). We also calculated π among chromosomes carrying the resistant or the susceptible allele of A2469G, and did not find altered patterns of diversity around *pst* (Figure S5, C and D, in File S1).

It is common to find components of the immune system where natural selection has driven rapid evolution of the protein sequence, which is normally interpreted as being caused by selection by pathogens (Obbard *et al.* 2009). To test whether this was the case for *pst*, we tested whether other amino acid variants had been fixed in *pst* using the MKT (McDonald and Kreitman 1991). *D. yakuba* and *D. simulans* sequences were used to infer the sequence of the most recent common ancestor of *D. simulans* and *D. melanogaster*. Analyzing polymorphisms from 165 lines from the DGRP panel and divergence from the most recent common ancestor of *D. simulans* and *D. melanogaster*, we found no signature of positive selection (low frequency variants excluded; synonymous polymorphism = 7, synonymous divergence = 13.23, nonsynonymous polymorphism = 13, nonsynonymous divergence = 32.46, α = 0.76, χ^2^ = 0.242, *P* = 0.625). Therefore, there is no evidence of positive selection on the amino acid sequence of Pastrel over the last ∼3 MY.

## Discussion

It has been argued that susceptibility to infectious disease may frequently have a simpler genetic basis than many other quantitative traits because natural selection drives major-effect resistance alleles up in frequency in populations (Hill 2012; Magwire *et al.* 2012). At first sight susceptibility to DCV in *Drosophila* would appear to be a clear example of this pattern, with a restriction factor called Pastrel explaining as much as 78% of the genetic variance in this trait (Cogni *et al.* 2016). However, we have found that this belies considerable complexity within this locus. Strikingly, in a sample of just seven alleles from natural populations, we found four phenotypically distinct allelic classes conferring differing levels of resistance to DCV. Furthermore, both coding and *cis*-regulatory variants in *pst* control resistance. The coding sequence variant that we characterized appears to be an old polymorphism that has been maintained at a relatively stable frequency, possibly as a result of balancing selection.

As we have found for DCV, it may be common for genes affecting quantitative traits to have multiple alleles (allelic heterogeneity). The most important gene controlling resistance to the sigma virus DMelSV in *D. melanogaster* is *CHKov1*, where there are three alleles conferring differing levels of resistance (Magwire *et al.* 2011). Increased transcription of the detoxification gene *Cyp6g1* confers resistance to insecticides in *D. melanogaster*, and again there are multiple alleles segregating in nature (Schmidt *et al.* 2010). One of the most-studied genes in natural populations of *Drosophila* is *Adh*, and multiple alleles are associated with ADH activity (Laurie and Stam 1994; King *et al.* 2012b). Allelic heterogeneity may not be restricted to these genes with large phenotypic effects. In a recent study on gene expression in *D. melanogaster*, 7922 expression QTL (eQTL) were mapped, and allelic heterogeneity was found in 95% of eQTLs acting in *cis* and 78% of eQTLs acting in *trans* (King *et al.* 2014).

An amino acid polymorphism in Pastrel is the most important factor determining susceptibility to DCV. There are multiple lines of evidence to support this. First, this is the only genetic variant that can explain the largest changes in resistance that we see in two large genetic mapping experiments. Second, when populations have been artificially selected for DCV resistance, this site shows the largest increase in frequency in the entire genome (Martins *et al.* 2014). Finally, we verified the phenotypic effect of this site by modifying it in transgenic flies. These transgenic flies were generated by inserting large BACs that contained a copy of the *pastrel* gene along with upstream sequences. We had intended to combine this with a mutant allele of *pastrel*, but this cross failed, possibly because the BAC failed to complement the mutation or due to other recessive lethal alleles on the chromosome. We therefore assayed the effect of this construct in flies that had a hemizygous wild-type-susceptible allele. This may be one of the reasons why the transgenic flies did not have as large an effect on resistance as was seen in the association studies. In the future, genome editing with *Cas9* will allow the gene to be modified seamlessly in its natural location in the genome.

The ancestral state at this site was the susceptible allele threonine. Three other major-effect polymorphisms that affect susceptibility to viruses in *Drosophila* have been identified at the molecular level, and in all cases the ancestral state was susceptible (Bangham *et al.* 2008; Magwire *et al.* 2011; Cao *et al.* 2016). This fits with a model whereby genetic variation is arising because there is continual input of novel resistance alleles into populations from mutations, and these are then favored by natural selection.

Resistance could evolve by improving existing antiviral defenses or by altering the myriad of host factors hijacked by the virus for its own benefit. For example, in *C. elegans*, susceptibility to the Orsay virus is determined by a polymorphism that disables the antiviral RNAi defenses (Ashe *et al.* 2013), while bacteriophage resistance is frequently associated with changes to surface receptors used by the virus to enter cells (Longdon *et al.* 2014). In a previous study, we found that knocking down the susceptible allele of *pst* makes flies even more susceptible (Magwire *et al.* 2012), while in this study we found that overexpressing the susceptible allele makes flies resistant. Therefore, the threonine to alanine mutation that we observe in *pst* is an improvement to an existing antiviral defense.

Patterns of genetic variation at the *pst* locus are complex. We found extensive structural variation, with multiple duplications and deletions of the gene present in natural populations. Gene duplications and rearrangements frequently affect gene function and can be evolutionarily important. However, these variants are not annotated in the standard versions of the DGRP or DSPR panels, and it took a considerable amount of laboratory work and analysis to characterize this variation. This reflects the difficulty of identifying structural variation using short-read sequencing, and represents a limitation of these genomic resources. In this case, however, we did not find a significant association between these structural variants and resistance to DCV.

There is also genetic variation in the expression of *pst*. There was a single QTL that controls *pst* expression centered on *pst* itself, suggesting that *cis*-regulatory variants control *pst* expression. Higher levels of *pst* expression are associated with increased resistance to DCV. This is unsurprising, as when we have experimentally altered *pst* expression by RNAi or by overexpressing the gene, DCV resistance is altered. Both SNPs and structural variants in the region are associated with *pst* expression. However, the *cis*-regulatory variants which are causing increased expression could not be unambiguously identified because of LD between these sites. Interestingly, the structural variants themselves were not significantly associated with survival after DCV infection, perhaps suggesting that they are not the main cause of variation in gene expression. Nonetheless, given the central role this gene plays in antiviral defense, it is tempting to speculate that these complex structural changes may have had some functional role, perhaps against other viruses (or we may simply lack the statistical power to detect effects on DCV) (Martins *et al.* 2014).

Why is genetic variation in susceptibility to DCV maintained in populations? There is likely to be selection favoring alleles that increase resistance in natural populations because DCV is the most virulent virus that has been isolated from *Drosophila* and field studies have found it to be geographically widespread (Christian 1987) [although recent surveys have suggested that it may have a low prevalence (Webster *et al.* 2015)]. *pastrel* has also been implicated in resistance to other viruses related to DCV (Martins *et al.* 2014). Given that the resistant allele is likely to enjoy a selective advantage, an important question is why the susceptible alleles have not been eliminated by natural selection. To understand how selection has acted on the amino acid variant that causes resistance, we examined geographical variation in its frequency and patterns of LD with neighboring sites. We could detect no evidence of natural selection causing changes in allele frequency through time or space. This is in stark contrast to the partial selective sweeps that we have seen in the two other major-effect polymorphisms affecting virus resistance (Bangham *et al.* 2008; Magwire *et al.* 2011). These polymorphisms are in the genes *CHKov1* and *P62* [*ref*(*2*)*P*] and both confer resistance to the sigma virus. In both cases the resistant allele has recently arisen by mutation and has spread through *D. melanogaster* populations under strong directional selection. In comparison to these polymorphisms it is clear that the polymorphism in *pst* is relatively old and does not show the same signatures of strong recent selection.

Observed population genetic patterns suggest that either the polymorphism has been evolving neutrally, or it has been maintained by balancing selection due to the benefits of resistance being balanced by harmful pleiotropic effects of the resistant allele on other traits. Long-term balancing selection can leave a signature of high divergence between the two alleles and elevated sequence polymorphism (Charlesworth 2006), but we have been unable to find any evidence of this in *pst*. This is not unexpected because the large effective population size of *D. melanogaster* means that LD declines rapidly around *pst*, and this is expected to erode any signature of balancing selection (Charlesworth 2006). A very similar pattern of sequence variation was recently reported around a polymorphism in the antimicrobial peptide Diptericin which affects susceptibility to bacterial infection (Unckless *et al.* 2016). This amino acid polymorphism is also found in the sibling species *D. simulans*, strongly suggesting it is maintained by balancing selection. Therefore, we cannot distinguish balancing selection and neutral evolution. While it seems likely that a polymorphism with such a large phenotypic effect is the target of natural selection, we would need additional data from natural populations to demonstrate that this was the case.

In *Drosophila*, increased resistance against bacteria and parasitoid wasps is associated with reduced fecundity and larval survival (Kraaijeveld and Godfray 1997; McKean *et al.* 2008). However, when populations of flies were selected for DCV resistance there was no detectable decline in other components of fitness (Faria *et al.* 2015). Unfortunately, while it is clear the resistant allele of *pst* is not highly costly, this negative result is hard to interpret. First, if the benefits of DCV resistance in nature are small, then a small cost that cannot be detected in the laboratory will be sufficient to maintain the polymorphism. Without having an estimate of the harm flies suffer due to DCV infection in nature it becomes impossible to reject the hypothesis that the benefits of resistance are balanced by pleiotropic costs. Second, costs of resistance are typically only expressed in certain environments and may affect many different traits (Kraaijeveld and Godfray 1997; McKean *et al.* 2008). It is possible that costs may not be detected if they are measured in the “wrong” environment or the trait affected is not measured—for example, it may increase susceptibility to other pathogen genotypes.

The function and identity of viral restriction factors in invertebrates remains poorly understood, and the mechanism by which Pastrel protects flies against DCV is unknown. This contrasts with vertebrates where a diverse range of restriction factors have been characterized that inhibit all steps of viral infection (see Yan and Chen 2012 for review). Studying natural variation in susceptibility to viral infection is proving a powerful way to identify novel restriction factors in *Drosophila* (Bangham *et al.* 2008; Magwire *et al.* 2011; Cao *et al.* 2016), and future work on these genes is likely to provide new insights into how invertebrates defend themselves against infection. One clue as to the function of Pastrel comes from its localization to lipid droplets in the larval fat body (Beller *et al.* 2006), as lipid droplets and lipid metabolism frequently play key roles in the viral replication cycle (Stapleford and Miller 2010). An alternative explanation is the reported involvement of Pastrel in the secretory pathway and Golgi organization (Bard *et al.* 2006).

We conclude that a single gene, *pastrel*, is the dominant factor that determines the susceptibility of *D. melanogaster* to DCV. This is a complex locus, with multiple alleles conferring different levels of resistance, with polymorphisms affecting both the expression and protein sequence of *pastrel* altering susceptibility to DCV. This gene has not been the target of strong directional selection, and the variation may be maintained by balancing selection. Overall, despite a single gene explaining most of the genetic variance in DCV susceptibility, this locus is remarkably complex.

## Supplementary Material

Supplemental material is available online at www.genetics.org/lookup/suppl/doi:10.1534/genetics.117.201970/-/DC1.
