# Supplementary material for: Complex Coding and Regulatory Polymorphisms in a Restriction Factor Determine the Susceptibility of Drosophila to Viral Infection
Source: Genetics. 2017 Jun 19;206(4):2159–73. doi: 10.1534/genetics.117.201970 (PMC5560813; doi:10.1534/genetics.117.201970)
Supplement: Supplementary file 1 [file 2159File002.docx]

| SNPs | Template | Polymorphism | PCR primers | *Galk* primers |
| --- | --- | --- | --- | --- |
| C398A | DGRP-358 | C | Fwd 5’ ACCTTCAGCCGGATTTTTCT 3’  Rev 5’ GCACAACTCCATTAGCAGCA 3’ | Fwd 5’ CAATTGACAGAGTTTGCCCCTTAGCACGTCATGTTGTTCATG  GGACAACCTGTAAGCCTGTTGACAATTAATCATCGGCA3’  Rev 5’ GAGATAATCTGGGATCTGTAAGTACTGCTACTGCGCATTAG  AATTTTGAATTCAAAGTCAGCACTGTCCTGCTCCTT 3’ |
| G484A | DGRP-358 | G | Fwd 5’CGTTTCGTCAGTTACCACCA 3’  Rev 5’ GCACAACTCCATTAGCAGCA 3’ | Fwd 5’ GCTCATTATCTGCGTTGACCTTCAGCCGGATTTTTCTGTCTTT  TTCAGTTTCCGCCTGTTGACAATTAATCATCGGCA 3’  Rev 5’ GTCCCATGAACAACATGACGTGCTAAGGGGCAAACTCTGTC  AATTGATTCGTGATTCTCAGCACTGTCCTGCTCCTT 3’ |
| A1870G | DGRP-370 | A | Fwd 5’ CTTAGCTCCAAGGCGAACAC 3’  Rev 5’ CACGCACAGCGTCTACAACT 3’ | Fwd 5’ GCCTTGCATCCGAACGGTCTCCTTGGCAGCCTTGTCAAAGGT  TCGCCTGATGGCCTGTTGACAATTAATCATCGGCA3’  Rev 5’ CCTTTCTAATAGGCAGCGGTAAAAAACGTTCAATGAGATAC  ATTCTGTCGTAAACAAGAGTCAGCACTGTCCTGCTCCTT3’ |
| A2469G | DGRP-370 | A | Fwd 5’ GCCGATACATTTCGGACACT 3’  Rev 5’ TCAGGTTTCCCGTCTAATCG 3’ | Fwd 5’ GTTGATATAAAGAACTTTAGCACCGAGGTGAGGTCTTCCAG  CATGGTGTCCATGCCTGTTGACAATTAATCATCGGCA 3’  Rev 5’ CTAATCAACATCATGGCCAATAAGCTTGATCCAAAAGTCTT  TGACCATATAATGCTATTGTCAGCACTGTCCTGCTCCTT 3’ |
| TA2911(2)CC | DGRP-21 | CC | Fwd 5’ CCCTGCGGATGTTTATGTCT 3’  Rev 5’ GGCGGTGTTGTTTTCGTACT 3’ | Fwd 5’CTGTGTTCGAAAACCGGAAATCAGAACCATTCCGGTTCAAAATTCTCCTTTT  CCTGTTGACAATTAATCATCGGCA 3’  Rev 5’GGATAACTTACTTAATATGAGCACAAATTGACAAAGATGAAATTGTTTTAGC  TCAGCACTGTCCTGCTCCTT 3’ |

Table S1 List of fly lines and primers used in amplifying *pst* alleles containing different SNPs and *galK*. For *galk* primers, sequences underlined are homologous sequences to the genome that flank the target region.

| Name | Sequence | SNP/variant amplified |
| --- | --- | --- |
| TA78(79)GG_f1.2 | CTTTTATTACAATTATTTCAACAGGGTTACTA | TA2911(2)CC **TA** |
| TA78(79)GG_f2.1 | AATTATTTCAACAGGGTTACGG | TA2911(2)CC **GG** |
| TA78(79)GG_r1.2 | CTTCATGTCCCTGAATCCAAA | TA2911(2)CC **TA** |
| TA78(79)GG_r2.2 | ACCATGCTGGAAGACCTCAC | TA2911(2)CC **GG** |
| C521T_f1.1 | GCATGGTGTCCATGAAGAC | A2469G **G** |
| C521T_f2.1 | GCATGGTGTCCATGAAGAT | A2469G **A** |
| C521T_r1.1 | TCCTCGACAGGAACCCAGTA | A2469G **G** |
| C521T_r2.2 | CCGATGGCAAAGGATTTTT | A2469G **A** |
| T2506C_f1.1 | CAGTTTCCGTTGCAGTTCT | A1870G **A** |
| T2506C_f2.5 | CTCACCGCTCCCACCTCTT | A1870G **G** |
| T2506C_f2.7 | CCCTGACCAAGCGGTAAATA | A1870G **G** |
| T2506C_r1.2 | GGCGAATAGATTAGGAAACCAT | A1870G **A** |
| T2506C_r2.3 | GCGTCCGTGAGTCATCCTAA | A1870G **G** |
| Pst 1F Fwd | CTGTGTTCGAAAACCGGAAATC | *pst* gene |
| Pst 2989R Rev | CATTTTGGGTTTAGCACGCTAG | *pst* gene |
| Pst_2c_brkpnt_F | AGCGTGCTAAACCCAAAATG | Existing of duplication of *pst* (variant 3) |
| Pst_2c_brkpnt_R | TGAACCGGAATGGTTCTGAT | Existing of duplication of *pst* (variant 3) |
| Pst_TD1_F | GCAGGAGTACTGGTGGACCT | Existing of duplication of 7960bp (variant 1) |
| Pst_TD1_R | GTCATACCGAAGCCTGAACC | Existing of duplication of 7960bp (variant 1) |

Table S2 Diagnostic primers amplifying different *pst* SNPs and verifying breakpoint of structure variants. SNP(s) amplified by the primers are underlined

**
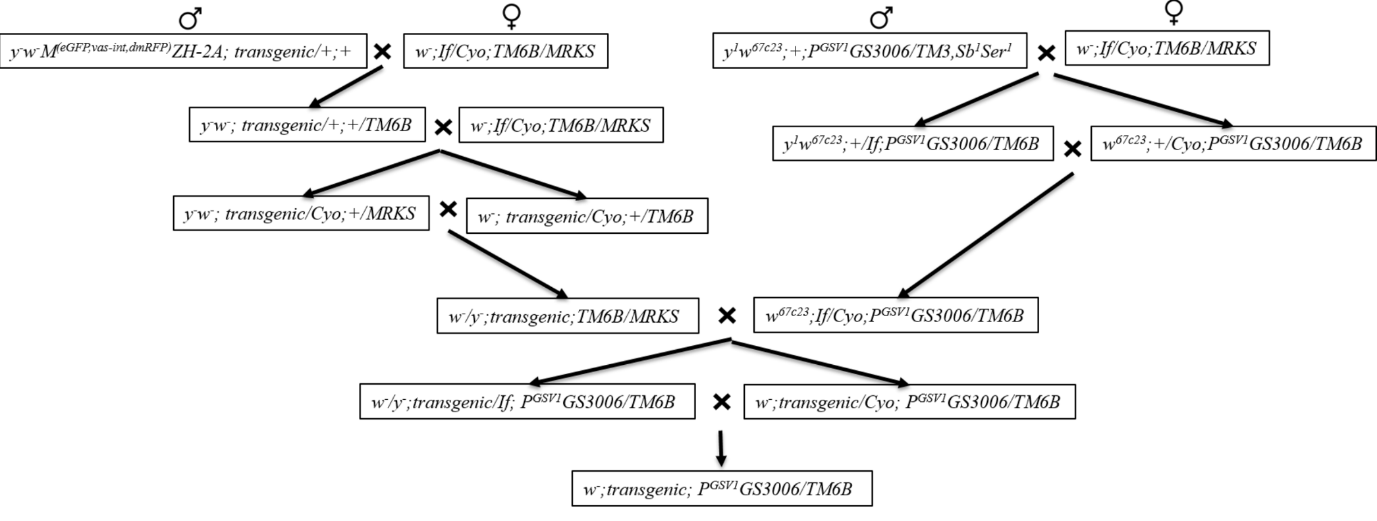
**

**Figure S1 *Drosophila* crossing scheme.**

**
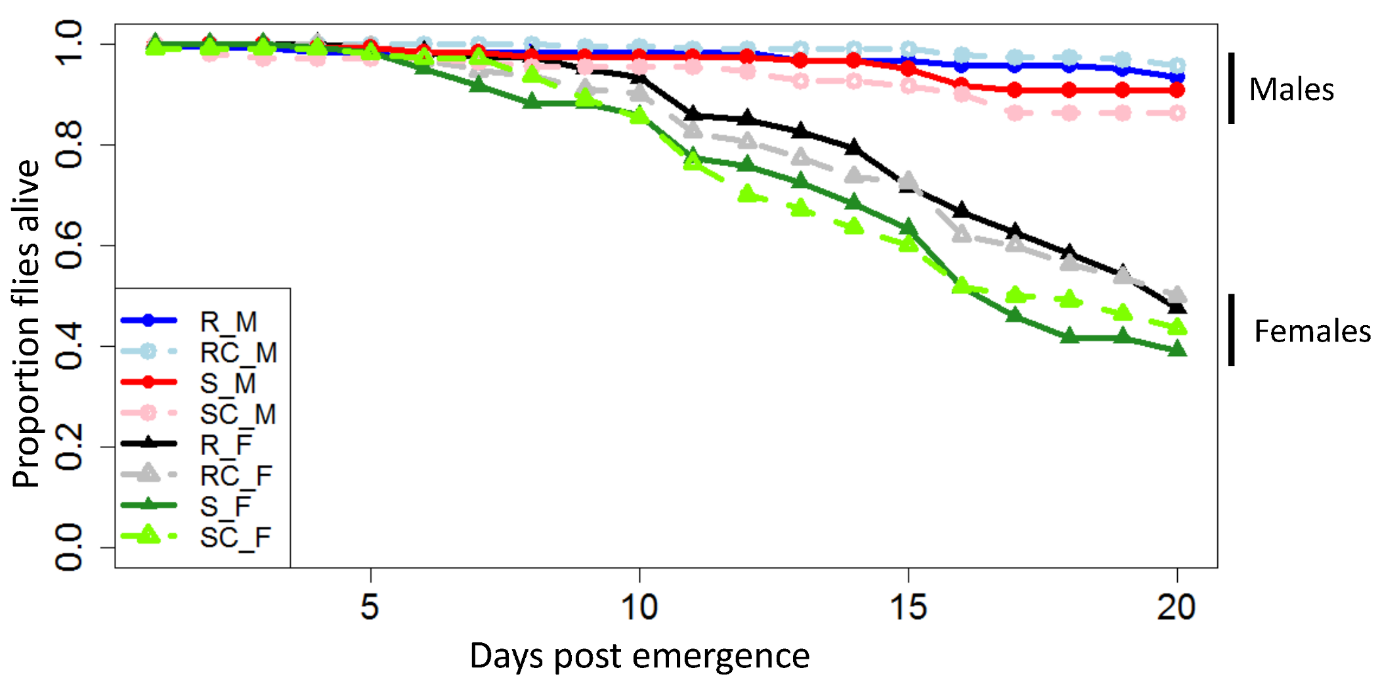
**

**Figure S2 Survival of flies overexpressing different pst A2469G alleles.** Fly mortality was recorded daily for 20 days post emergence. “R” stands for flies overexpressing resistant *pst* allele “G”, “S” stands for flies overexpressing susceptible *pst* allele “A”. “F” and “M” represent females and males separately. “RC” and “SC” are controls for overexpression resistant and susceptible *pst* allele separately. The flies are not infected by DCV.

**
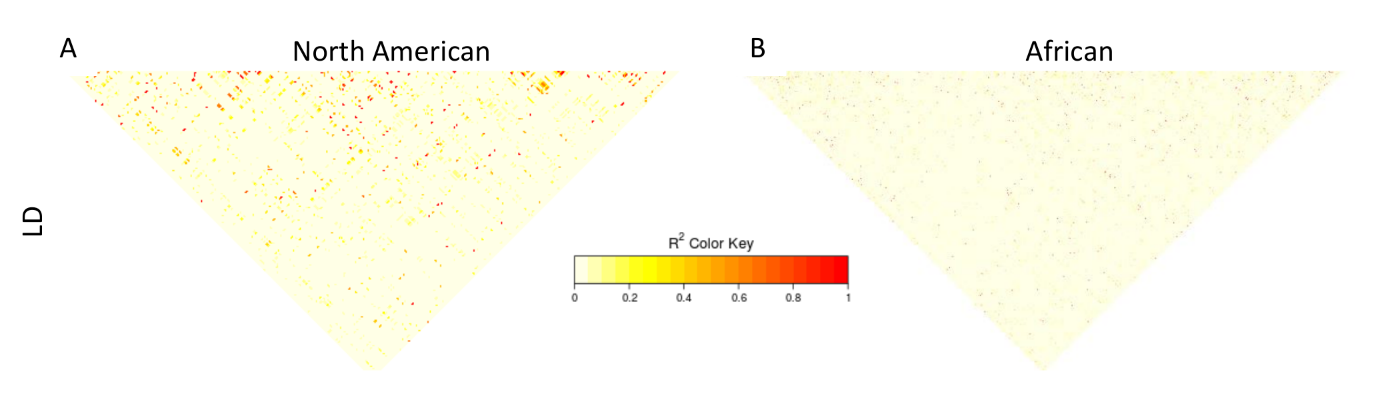
**

**Figure S3 Linkage diseuilibrium around *pst***. Heat maps of linkage disequilibrium in a 20kb region around *pst* in a North American population (DGRP; panel A) and African population (DPGP2; panel B), showing pairwise estimates of *R^2^*.

**
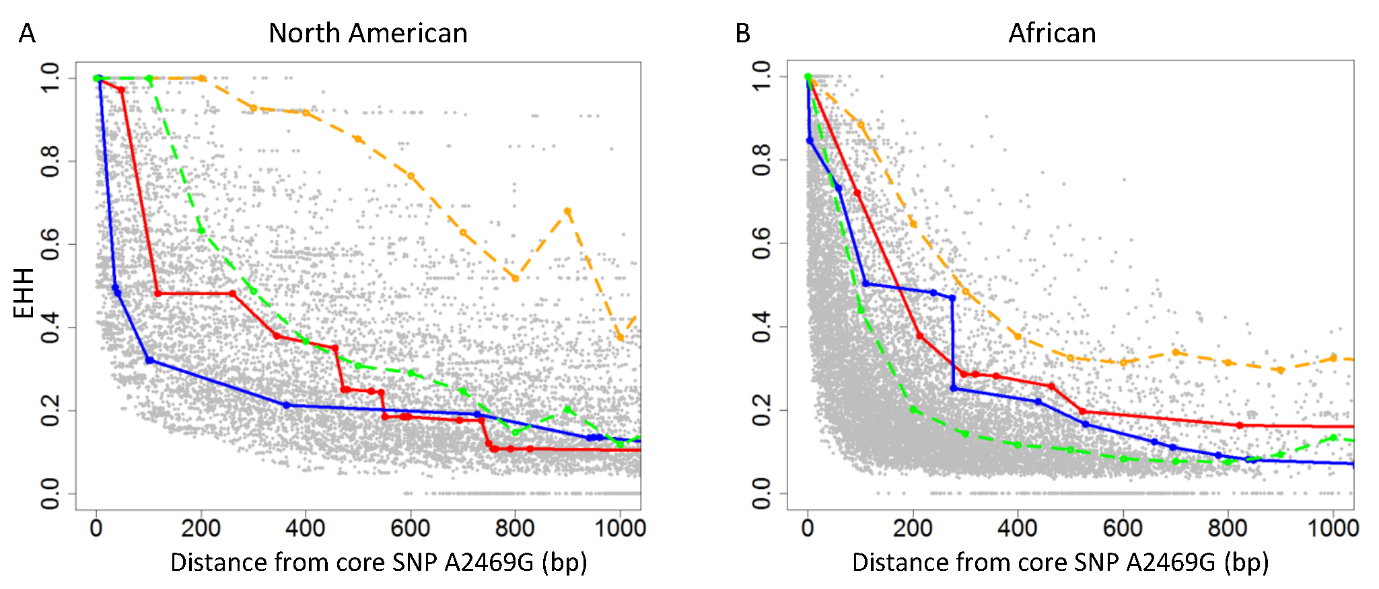
**

**Figure S4 Extended haplotype heterozygosity (EHH) of ancestral (susceptible) allele of the core SNP A2469G.** Breakdown of extended haplotype heterozygosity (EHH) over distance between the ancestral (susceptible) allele of the core SNP A2469G and SNPs within the distance of 1000 bases from the mutation. Panel (A) is North American population and (B) is African population. Red line and blue line are EHH breakdown of upstream and downstream of SNP A2469G respectively. The grey points are a null distribution generated by calculating the EHH using other SNPs that are a similar frequency in the region as the core. The orange dash line indicates top 5% EHH value of this null distribution while green dash line indicates median EHH.


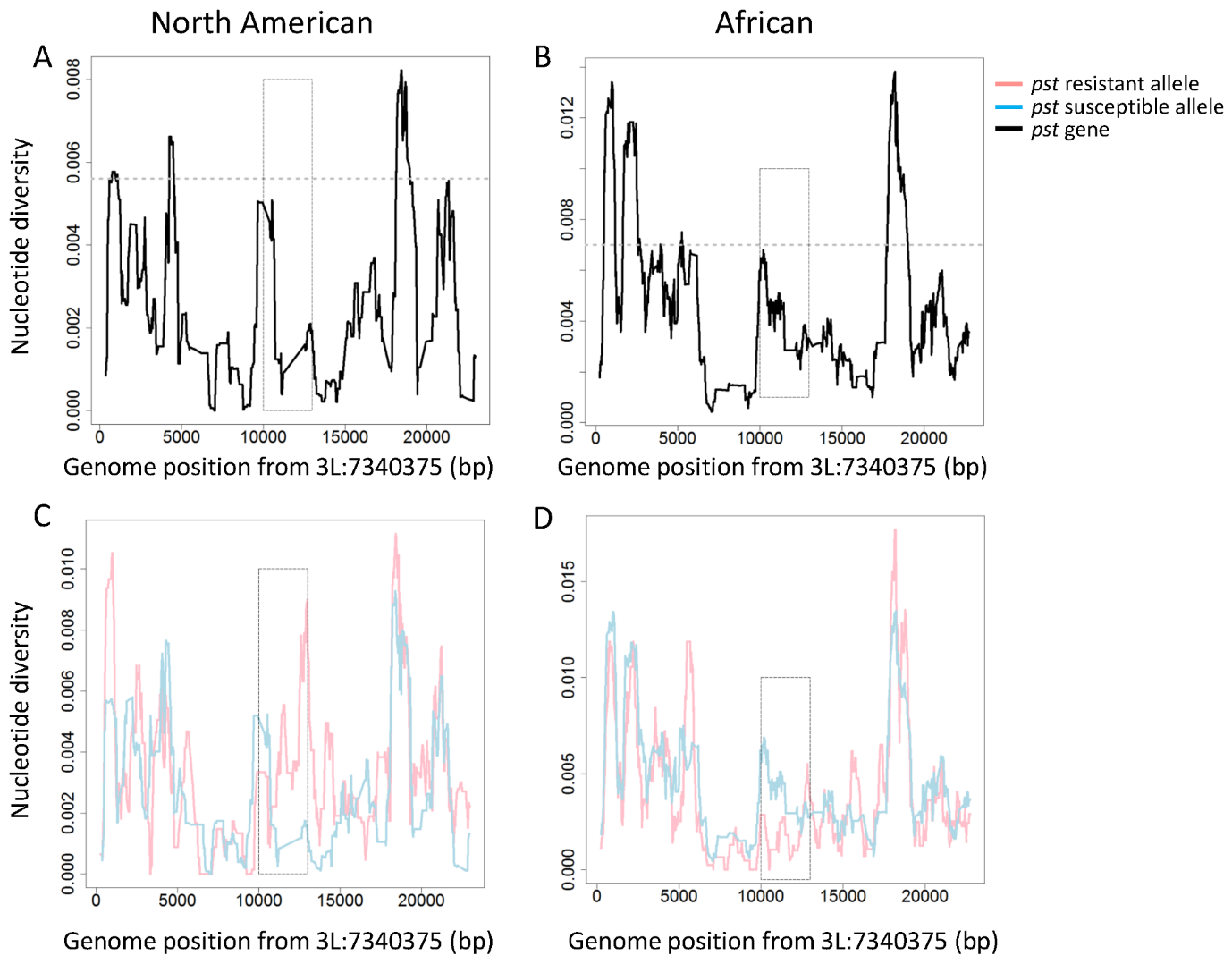


**Figure S5 Nucleotide diversity of region 3L: 7340375-7363363 containing *pst* gene.** Panels (A) and (B) are the nucleotide diversity (π) in the North American (DGRP) and Zambian populations respectively. Dashed lines are the average π over the entire genome. Panels (C) and (D) are the nucleotide diversity of chromosomes carrying the resistant allele or the susceptible allele of A2469G in the North American (DGRP) and Zambian populations respectively. Red lines represent chromosomes with the resistant allele “G” for SNP A2469G and blue lines represent the susceptible allele. Dashed boxes indicate the *pst* gene.
